# Tumor Burden Score Stratifies Prognosis of Patients With Intrahepatic Cholangiocarcinoma After Hepatic Resection: A Retrospective, Multi-Institutional Study

**DOI:** 10.3389/fonc.2022.829407

**Published:** 2022-03-07

**Authors:** Hui Li, Rongqiang Liu, Haizhou Qiu, Yang Huang, Wenbin Liu, Jiaxin Li, Hong Wu, Genshu Wang, Dewei Li

**Affiliations:** ^1^ Department of Hepatobiliary Pancreatic Tumor Center, Chongqing University Cancer Hospital, School of Medicine, Chongqing University, Chongqing, China; ^2^ Department of Liver Surgery & Liver Transplantation, State Key Laboratory of Biotherapy and Cancer Center, West China Hospital, Sichuan University, Chengdu, China; ^3^ Department of Hepatic Surgery and Liver Transplantation Center, the Third Affiliated Hospital of Sun Yat-sen University, Guangzhou, China

**Keywords:** tumor burden score, CA19-9, intrahepatic cholangiocarcinoma, hepatectomy, prognosis

## Abstract

**Background:**

The prognostic significance of tumor burden score (TBS) on patients who underwent curative-intent resection of intrahepatic cholangiocarcinoma (ICC) has not been evaluated. The present study aimed to investigate the impact of TBS and its synergistic effect with CA19-9 (combination of TBS and CA19-9, CTC grade) on long-term outcomes.

**Methods:**

Patients who underwent radical resection of ICC between 2009 and 2017 were retrospectively identified from a multi-center database. The overall survival (OS) and recurrence-free survival (RFS) were examined in relation to TBS, serum preoperative CA19-9, and CTC grade.

**Results:**

A total of 650 patients were included in our study (509 in the derivation cohort and 141 in the validation cohort). Kaplan–Meier curves showed that both TBS and CA19-9 levels were strong predictors of survival outcomes. Patients with elevated TBS grade or elevated CA19-9 were associated with worse OS and RFS (both p < 0.001). As expected, CTC grade also performed well in predicting long-term outcomes. Patients with low TBS/low CA19-9 (CTC grade 1) were associated with the best OS as well as RFS, while high TBS/high CA19-9 (CTC grade 3) correlated to the worst outcomes. In the validation cohort, TBS grade, preoperative CA19-9, and CTC grade also stratified prognosis among patients (p < 0.001 for each).

**Conclusions:**

Both tumor morphology (tumor burden) and tumor-specific biomarker (serum CA19-9) were important when evaluating prognosis of patients with resectable ICC. Serum CA19-9 and TBS showed a synergistic effect on prognostic evaluation. CTC grade was a promising tool in stratifying prognosis of ICC patients after curative resection.

## Introduction

Liver cancer is one of the most commonly diagnosed malignancies and the third leading cause of cancer death worldwide (1). There were approximately 906,000 new cases of liver cancer and 830,000 liver cancer deaths worldwide in 2020 (1). Intrahepatic cholangiocarcinoma (ICC) is cholangiocellular carcinoma originated from intrahepatic bile duct cells, accounting for 5%–20% of primary liver malignancies (2). The risk factors of ICC mainly include intrahepatic bile duct stones, hepatitis B or C infection, primary sclerosing cholangitis, hepatobiliary parasites, and diabetes (3, 4). The incidence of ICC demonstrates a geological difference, with the highest incidence rate in Asia, while a noticeable increase was also observed in western countries in recent years (5, 6). At present, the therapeutic strategies for ICC mainly depend on surgical resection and liver transplantation, supplemented by chemotherapy and immunotherapy treatment (7). However, due to its high degree of malignancy, ICC usually invades and metastasizes in the early stages. As a result, less than 15% of patients are eligible for radical resection (8). In addition, even for surgically treated ICC, the long-term outcome remains unsatisfactory due to rapid progression and frequent incidence of recurrence, with a 5-year survival rate ranging from 30% to 35% (9). Improving ICC patient prognosis has remained a medical challenge. Currently, many biomarkers have been reported to predict the prognosis of ICC patients, but their clinical applications have been very limited (10). Thus, there is an urgent need for a simple and effective marker for better prediction of ICC patient prognosis, so clinicians can implement individualized treatment at the earliest to improve patient survival.

Tumor burden score (TBS), a new metric based on tumor size and tumor number, was firstly proposed for patients with colorectal liver metastases in 2017 (11). Recently, studies demonstrated that TBS also showed promising utility in stratifying prognosis of patients with hepatocellular carcinoma who underwent hepatectomy or liver transplantation (12–14). However, whether the prognosis of ICC patients could be dictated by TBS remained unclear. Thus, this study aimed to investigate the prognostic significance of TBS grade in surgically treated ICC patients.

Carbohydrate antigen 19-9 (CA19-9), a carbohydrate-related protein, was conventionally used for diagnosis and prognostic evaluation of pancreatic cancer, colorectal cancer, gastric cancer, and cholangiocarcinoma (15, 16). Previous studies have reported that elevated preoperative serum CA19-9 was associated with poor prognosis of ICC patients and could serve as an effective biomarker (17, 18). In the present study, we explored the correlation between serum CA19-9 levels and long-term survival outcomes of ICC patients. Furthermore, we proposed a novel index by combination of TBS grade with CA19-9 grade (CTC grade) and analyzed its prognostic effect on predicting patient survival. The CTC grade demonstrated a promising accuracy in predicting tumor relapse as well as patient survival and can stratify ICC patients into groups with different long-term outcomes.

## Materials and Methods

### Patients

This study was carried out in accordance with the Declaration of Helsinki and approved by the Ethics Committees of relevant institutions (19). We retrospectively collected and reviewed 745 consecutive ICC patients who underwent radical resection at West China Hospital, the Third Affiliated Hospital of Sun Yat-sen University and Chongqing University Cancer Hospital from 2009 through 2017. The exclusion criteria were as follows: patients with extrahepatic metastasis, patients with other tumors, recurrent tumors, patients who underwent liver transplantation, the status of surgical margin was positive, or patients with local organ invasion. Patients who passed the exclusion criteria were selected and reviewed for the clinicopathologic characteristics and perioperative outcomes. All patients or their relatives signed a consent form.

### TBS Definition and CTC Grade Evaluation

As previously described, TBS is defined as the distance from the origin of the Cartesian plane and include two variables: maximum tumor size (*x*-axis) and number of tumors (*y*-axis) (11). Its calculation formula is based on the Pythagorean theorem principle: TBS^2^ = (maximum tumor diameter)^2^ + (number of tumors)^2^. For each patient, the maximum tumor diameter and the number of tumors were obtained from preoperative imagological examination, confirmed by postoperative pathology. Then, TBS was calculated by the above formula.

The cutoff value for CA19-9 was 37 U/ml based on ELISA assays. A 2-year survival was set as endpoint, and receiver-operating characteristic (ROC) curves were applied to determine the optimal cutoff value of TBS as Youden index attained maximum value. Patients were classified into high and low grade according to the cutoff values. Subsequently, CTC grade was grouped based on TBS grade and CA19-9 grade. In brief, patients with both low CA19-9/low TBS grade were categorized into CTC grade 1, those with either high CA19-9/low TBS grade or low CA19-9/high TBS grade were categorized into CTC grade 2, and those with both high CA19-9/high TBS grade were categorized into CTC grade 3.

### Follow-Up

Patients were followed according to National Comprehensive Cancer Network, with regular conventional tumor markers and contrast-enhanced ultrasonography each month at first half year, then every 3 months for 2 years, and every half year thereafter. Those who did not come back to the hospital for reexamination were subject to telephone follow-up survey. Patient survival data mainly included overall survival (OS) and recurrence-free survival (RFS). OS was calculated from hepatic resection to death or the last follow-up. RFS was considered as the time from the first operation to the earliest evidence of recurrence or the last follow-up.

### Statistical Analysis

The Student’s *t*-test or Wilcoxon rank sum test was used for quantitative data. Categorical variable was presented as number (percentage) and analyzed by chi-square or Fisher’s exact test, as appropriate. We used ROC curve to compare the prognostic value among different indicators. The Kaplan–Meier curve was plotted to describe OS and RFS, their differences were tested by log-rank test. Univariate and multivariate Cox regression models were employed to identify independent prognostic risk factors. Those clinicopathological parameters with p < 0.2 in the univariate analyses were selected in multivariate analyses, whereas TBS grade and CA19-9 grade were excluded from multivariate analyses to avoid collinearity bias (20). All data were performed by SPSS (version 23.0, Chicago, IL, United States) and MedCalc (version 20.0.3.0, Ostend, Belgium). *p* **-**value less than 0.05 was considered as statistically meaningful.

## Results

### Patient Population

**Figure 1:**
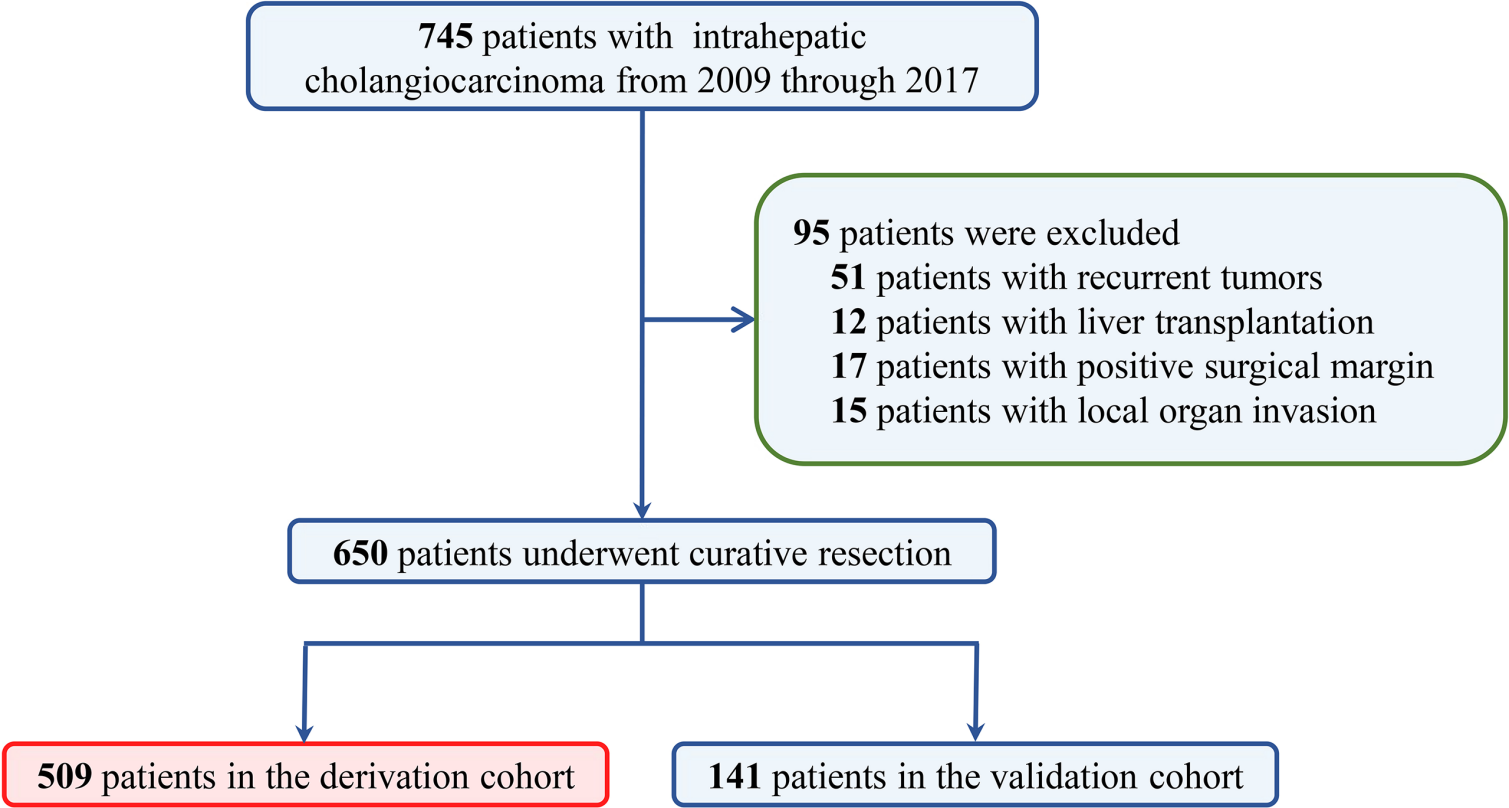
The selection process of ICC patients in the final analysis. ICC, intrahepatic cholangiocarcinoma.

A total of 745 ICC patients who underwent curative-intent hepatectomy between 2009 and 2017 were retrospectively reviewed. Among them, 95 were excluded because of recurrent tumors, local organ invasion, positive surgical margin, or liver transplantation (**Figure 1**). Finally, 650 patients who underwent curative resection were included (509 from West China Hospital in derivation cohort and 141 from the Third Affiliated Hospital of Sun Yat-sen University and Chongqing University Cancer Hospital in validation cohort). As summarized in **Table 1**, 321 (49.4%) were male and most patients were older than 50 (476, 73.2%). A total of 392 (60.3%) patients were associated with tumors larger than 5 cm and 454 (69.8%) were solitary tumor. A total of 242 (37.2%) patients had normal preoperative CA19-9 value. Grouped by TBS, 213 (32.8%) were in the low TBS grade group.

**Table 1:** Baseline characteristics of 650 ICC patients who underwent curative resection.

Variables	All patients (*n* = 650)	Derivation cohort (*n* = 509)	Validation cohort (*n* = 141)	*p*-value
Age, ≤50/>50	174/476	135/374	39/102	0.830
Gender, male/female	321/329	249/260	72/69	0.704
HBsAg, +/-	192/459	149/360	42/99	0.917
Hepatolithiasis, +/-	94/556	72/437	22/119	0.786
Tumor size, <5/≥5	258/392	207/302	51/90	0.381
Tumor number, 1/2/≥3	454/135/61	354/110/45	100/25/16	0.322
Differentiation, well/moderate-poor	26/624	19/490	7/134	0.627
Capsular invasion, +/-	418/232	327/182	91/50	1.000
MVI, +/-	61/589	50/459	11/130	0.518
Node invasion, +/-	151/499	122/387	29/112	0.432
Perineural invasion, +/-	88/562	73/436	15/126	0.270
Cirrhosis, +/-	175/475	140/369	35/106	0.592
TNM stage, I–II/III	187/463	147/362	40/101	0.917
CA19-9, <37/≥37	242/408	187/322	55/86	0.624
TBS grade, low/high	213/437	168/341	45/96	0.840
CTC grade, 1/2/3	98/259/293	73/209/227	25/50/66	0.399

ICC, intrahepatic cholangiocarcinoma; MVI, microvascular invasion; TNM, tumor-node-metastasis; CA19-9, carbohydrate antigen 19-9; TBS, tumor burden score; CTC, combination of TBS and CA19-9 grade.

### Association Between CTC Grade and Clinicopathologic Features

Among 509 patients in the derivation cohort, 73 (14.3%) patients were stratified into CTC grade 1, 209 (41.1%) patients in CTC grade 2, and 227 (44.6%) in CTC grade 3 (**Table 2**). ROC curve identified an optimal cutoff value for TBS of 4.71. Mean TBS was 3.74 in CTC grade 1 and 5.86 in CTC grade 2. Apart from tumor size and tumor number, CTC levels were significantly correlated to liver capsular invasion (*p* = 0.015), microvascular invasion (*p* = 0.006), lymph node invasion (*p* = 0.015), and TNM stages (*p* = 0.013).

**Table 2:** Correlation between CTC grade and clinicopathological characteristics in derivation cohort.

Variables	CTC grade			*p*-value
	1 (*n* = 73)	2 (*n* = 209)	3 (*n* = 227)	
Age, ≤50/>50	18/55	54/155	63/164	0.830
Gender, male/female	34/39	98/111	117/110	0.578
HBsAg, +/-	19/54	63/146	65/160	0.807
Hepatolithiasis, +/-	9/64	42/167	31/196	0.125
Tumor size, <5/≥5	65/8	99/110	43/184	<0.001
Tumor number, 1/2/≥3	60/10/3	151/41/17	143/54/30	0.016
Differentiation, well/moderate-poor	4/69	8/201	7/220	0.315
Capsular invasion, +/-	39/34	128/81	160/67	0.015
MVI, +/-	4/69	13/196	33/194	0.006
Node invasion, +/-	12/61	42/167	68/159	0.015
Perineural invasion, +/-	12/61	28/181	33/194	0.801
Cirrhosis, +/-	22/51	62/147	56/171	0.444
TNM stage, I–II/III	27/46	69/140	51/176	0.013
TBS	3.74 (1.53)	5.86 (2.54)	7.52 (2.29)	<0.001

ICC, intrahepatic cholangiocarcinoma; MVI, microvascular invasion; TNM, tumor-node-metastasis; CA19-9, carbohydrate antigen 19-9; TBS, tumor burden score; CTC, combination of TBS and CA19-9 grade.

### Prognostic Implication of TBS, CA19-9, and CTC Grade

**Figure 2:**
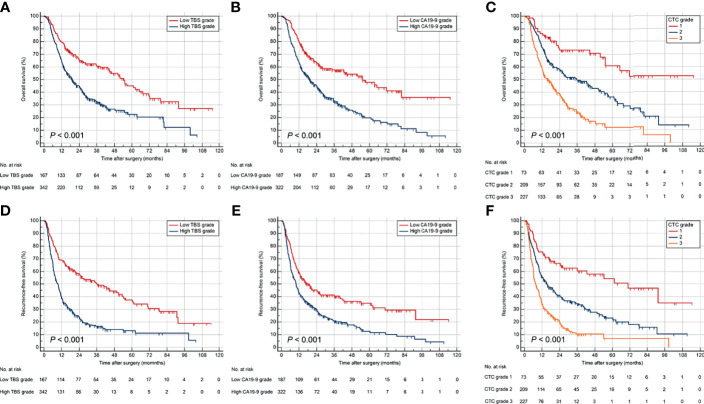
Kaplan–Meier curves for OS and RFS stratified by TBS **(A, D)**, CA19-9 **(B, E)**, and CTC grade **(C, F)** in the derivation cohort. OS, overall survival; RFS, relapse-free survival.

Stratifying the derivation cohort according to TBS seemed to provide effective discrimination of survival outcomes. Patients in high TBS group were associated with worse OS and RFS than those in low TBS group (**Figures 2A, D**). Preoperative CA19-9 level also showed a significant power in stratifying patients into groups with different survival outcomes. Patients with elevated preoperative CA19-9 had worse OS and RFS compared to those with normal values (**Figures 2B, E**). Importantly, the combination of TBS grade and CA19-9 (CTC grades), dividing patients into three risk groups, showed better prognostic discrimination power. Patients with low TBS grade as well as normal preoperative CA19-9 level showed the best survival, whereas those with high TBS grade/elevated CA19-9 level correlated to worst OS and RFS after curative resection (**Figures 2C, F**).

In the univariate Cox regression analysis, gender, hepatolithiasis, tumor size, tumor number, microvascular invasion (MVI), node invasion, perineural invasion, cirrhosis status, TNM stage, preoperative CA19-9 level, TBS grade, and CTC grade were characterized as potential factors affecting OS and subject to multivariate analyses. However, only MVI, node invasion, and CTC grade were identified as independent risk factors (**Table 3**). Similarly, the univariate Cox regression analysis of RFS showed that 8 of 16 clinicopathological parameters were potential factors, whereas only MVI, node invasion, and CTC grade were identified as independent risk factors (**Table 4**).

**Table 3:** Cox regression analysis for OS of ICC patients in the derivation cohort.

Variables	Univariate			Multivariate		
	HR	95% CI	*p*	HR	95% CI	*p*
Age, >50/≤50	1.130	0.870–1.466	0.359			
Gender, male/female	0.830	0.663–1.040	0.105	0.874	0.693–1.101	0.253
HBsAg, +/-	1.103	0.864–1.410	0.431			
Hepatolithiasis, +/-	1.348	1.016–1.788	0.038	1.320	0.987–1.765	0.062
Tumor size, ≥5/<5	1.277	1.013–1.609	0.039			
Tumor number	1.413	1.232–1.621	<0.001			
Differentiation, moderate-poor/well	1.149	0.703–1.878	0.580			
Capsular invasion, +/-	1.078	0.851–1.364	0.535			
MVI, +/-	1.745	1.245–2.445	0.001	1.435	1.013–2.033	0.042
Node invasion, +/-	2.348	1.844–2.991	<0.001	2.269	1.732–2.972	<0.001
Perineural invasion, +/-	1.643	1.138–2.092	0.005	1.340	0.979–1.834	0.068
Cirrhosis, +/-	0.803	0.628–1.027	0.081	0.867	0.668–1.093	0.226
TNM stage, III/I–II	1.317	1.020–1.700	0.035	1.032	0.775–1.373	0.068
CA19-9, ≥37/<37	2.071	1.607–2.669	<0.001			
TBS grade, high/low	2.061	1.588–2.676	<0.001			
CTC grade						
1	Ref.	Ref.	Ref.	Ref.	Ref.	Ref.
2	2.246	1.444–3.495	<0.001	2.193	1.406–3.421	<0.001
3	4.289	2.770–6.642	<0.001	4.055	2.610–6.301	<0.001

ICC, intrahepatic cholangiocarcinoma; MVI, microvascular invasion; TNM, tumor-node-metastasis; CA19-9, carbohydrate antigen 19-9; TBS, tumor burden score; CTC, combination of TBS and CA19-9 grade; OS, overall survival.

**Table 4:** Cox regression analysis for RFS of ICC patients in the derivation cohort.

Variables	Univariate			Multivariate		
	HR	95% CI	*p*	HR	95% CI	*p*
Age, >50/≤50	0.992	0.785–1.255	0.950			
Gender, male/female	0.897	0.731–1.100	0.297			
HBsAg, +/-	1.182	0.946–1.476	0.141	1.181	0.939–1.486	0.154
Hepatolithiasis, +/-	0.978	0.742–1.290	0.877			
Tumor size, ≥5/<5	1.487	1.201–1.840	<0.001			
Tumor number	1.538	1.355–1.746	<0.001			
Differentiation, moderate-poor/well	1.204	0.864–1.680	0.273			
Capsular invasion, +/-	1.194	0.961–1.484	0.110	0.775	0.511–1.175	0.230
MVI, +/-	1.991	1.459–2.717	<0.001	1.740	1.258–2.407	0.001
Node invasion, +/-	1.915	1.528–2.399	<0.001	1.653	1.268–2.157	<0.001
Perineural invasion, +/-	1.251	0.940–1.663	0.124	1.075	0.792–1.460	0.642
Cirrhosis, +/-	0.923	0.735–1.160	0.493			
TNM stage, III/I-II	1.400	1.106–1.772	0.005	1.292	0.807–2.066	0.286
CA19-9, ≥37/<37	1.675	1.341–2.092	<0.001			
TBS grade, high/low	2.425	1.906–3.085	<0.001			
CTC grade						
1	Ref.	Ref.	Ref.	Ref.	Ref.	Ref.
2	2.192	1.492–3.222	<0.001	2.252	1.527–3.321	<0.001
3	4.022	2.741–5.900	<0.001	3.820	2.590–5.633	<0.001

ICC, intrahepatic cholangiocarcinoma; MVI, microvascular invasion; TNM, tumor-node-metastasis; CA19-9, carbohydrate antigen 19-9; TBS, tumor burden score; CTC, combination of TBS and CA19-9 grade; RFS, recurrence-free survival.

**Figure 3:**
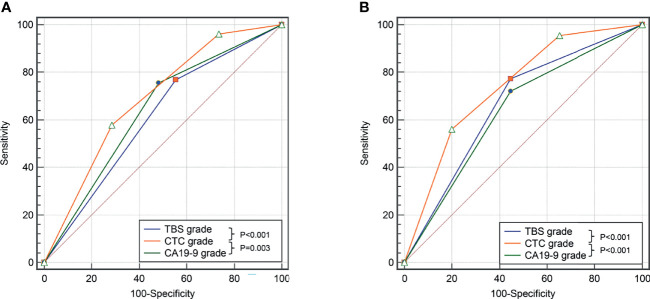
Comparison of the predictive value of TBS, CA19-9, and CTC grade in OS **(A)** and DFS **(B)** in the derivation cohort.

The distinguishing power of those independent risk factors in predicting prognostic outcomes were analyzed by the ROC method. The CTC grade showed the best discriminatory power in predicting OS as well as RFS of ICC patients after liver resection (area under curve [AUC] was 0.688 and 0.739, respectively) (**Figure 3** and **Supplementary Table 1**).

A total of 402 (61.8%) patients experienced tumor relapse within 2 years, namely, 319/509 (62.6%) in the derivation cohort and 83/141 (58.9%) in the validation cohort. A total of 57 (8.8%) patients experienced non-tumor-relapse-related death within 2 years. Kaplan–Meier curves showed that patients with elevated CTC grade were associated with worse OS and RFS within 2 years (**Supplementary Figure 1**). ROC demonstrated that CTC grade performed well in predicting 2-year OS and early recurrence (AUC was 0.675 and 0.728, respectively, **Supplementary Figure 2**).

### Validation of TBS Grade, CA19-9, and CTC Grade

**Figure 4:**
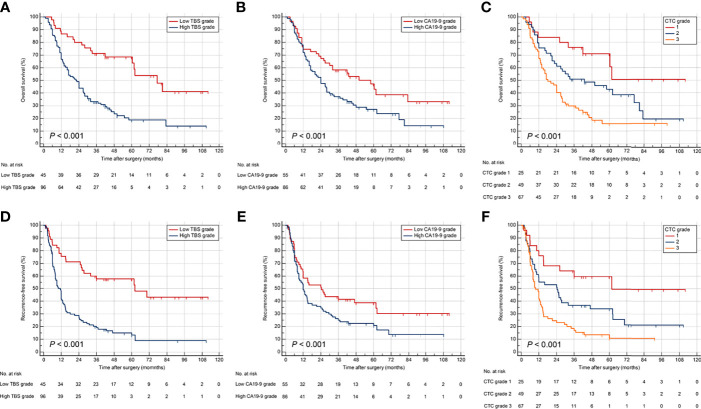
Kaplan–Meier curves for OS and RFS stratified by TBS **(A, D)**, CA19-9 **(B, E)**, and CTC grade **(C, F)** in the validation cohort.

The abilities of TBS grade, preoperative CA19-9, and CTC grade to stratify prognosis among ICC patients who underwent curative resection were validated in a cohort of 141 patients. The demographic and clinicopathological characteristics were comparable between validation and derivation cohort (**Table 1**). The CTC grade was significantly related to tumor size, tumor number, node invasion, and TNM stage in the validation cohort (**Supplementary Table 2**). Survival analyses verified TBS grade, preoperative CA19-9 level, and CTC grade as promising prognostic factors (**Figure 4**). In addition, Cox regression models for OS and RFS identified CTC grade as an independent risk factor in the validation cohort (**Supplementary Tables 3**, **4**).

## Discussion

ICC incidence has increased over the past decade, and radical resection remains the most effective treatment (6). Tumor staging is crucial to develop appropriate therapeutic strategies and to accurately evaluate the prognosis of ICC patients. Traditionally, ICC has been staged together with HCC under the category of ‘‘primary liver cancers” (21). Among traditional staging systems, like Barcelona Clinic Liver Cancer (BCLC) classification and Milan criteria, tumor size and number have been emphasized for their performance in prognostic stratification. The first internationally recognized staging system for ICC was proposed in the 7th edition of AJCC staging manual (22), in which tumor size was not included as a factor. In the current 8th edition of the AJCC staging system for ICC, T1 category was divided into T1a and T1b using a cutoff value of 5 cm in tumor size, underlining the effect of tumor size on outcomes of patients with solitary ICC, while T2 category was introduced to reflect the equivalent prognostic effect of tumor multifocality and vascular invasion (23). Nevertheless, there has been debate as to whether tumor size performs well in terms of prognostic stratification for ICC. Previous studies have indicated that tumor size could not serve as independent risk factor for prognostic prediction in ICC patients (24, 25), while Beetz et al. demonstrated that patients with “very early” ICC (based on tumor size alone) displayed a promising postoperative 5-year survival rate of 58.2% (26). Our previous studies also showed that a tumor size of 5 cm was not an independent prognostic predictor for surgically treated ICC patients, whereas multifocality was (27).

Among the 650 surgically treated ICC patients included in the present study, 392 (60.3%) patients had tumors > 5 cm, while 196 (30.2%) patients had multifocal ICCs. Both tumor size and number were identified as prognostic factors in univariate analyses. Thus, we suspected that an index combining tumor size and number may perform better in prognostic stratification for ICC. TBS, a recently developed measure that incorporates both tumor size and number in a continuous fashion, has been reported to be a valuable tool in evaluating prognosis of patients with colorectal liver metastasis and HCC (11, 28, 29). In this study, we used TBS to determine prognosis in surgically treated ICC patients and revealed that elevated TBS was associated with poor OS as well as RFS. A previous study has proposed a log-model Classification and Regression Tree-derived score, based on tumor size and number, and demonstrated that it was a valuable tool in evaluating prognosis of ICC patients (30). Our findings were consistent with those results, showing that tumor size and number-derived TBS performed well in prognostic stratification of ICC patients who underwent curative resection.

Apart from tumor morphology, serum CA19-9 has been recognized as a surrogate of cancer biology and is considered as a strong predictor of long-term outcomes among ICC patients (31, 32). Serum CA19-9 is a well-established biomarker for ICC and has been widely used in clinical practice to inform prognosis following therapies (31, 32). The present study validated the prognostic utility of preoperative serum CA19-9 and suggested that elevated CA19-9 level correlated to worse OS and RFS in ICC patients after curative resection. Incorporating tumor-specific biomarkers to prognostic index may improve the predictive accuracy and maximize clinical benefit by optimization of patient selection for surgical resection (33). Qiu et al. suggested that incorporating serum CA19-9 to a clinical index showed better value in prognostic stratification than CA19-9 alone (34). Thus, we proposed a novel index by combination TBS (tumor morphology) and CA19-9 (tumor-specific biomarker), termed CTC grade, and investigated its utility in predicting ICC patient long-term outcomes.

Of note, TBS and CA19-9 showed synergistic effect on prognosis stratification. Patients with high TBS/high CA19-9 grade (CTC grade 3) had worst OS and RFS, whereas individuals with low TBS/low CA19-9 grade (CTC grade 1) were associated with best prognosis. MVI and lymph-node invasion have been established as critical risk factors for poor OS and RFS in ICC (35, 36). In the present work, CTC grade demonstrated accurate predictive value in ICC patients following curative resection, better than MVI and lymph node invasion. In the future, studies could explore whether a combination of CTC grade with MVI or/and node invasion would further improve the prediction performance. The primary findings were validated in an external dataset. Collectively, the data from the present study highlighted the importance of both tumor morphology and serum biomarker in determining survival outcomes of patients who underwent curative resection of ICC. A novel index was proposed by combination tumor size, number, and serum CA19-9, showing promising predictive accuracy in prognosis. Surgeons should take preoperative CA19-9 into consideration when considering curative resection for high tumor burden ICCs.

Several limitations should be taken into consideration when interpreting our results. Owing to the retrospective nature, the current study may be subject to selection bias regarding which patients were offered surgical resection. Additionally, only patients undergoing hepatic resection (a portion of the overall population of ICC patients) were included in this study; the findings were strict to surgically treated patients rather than to the overall ICC population. Furthermore, while the multi-centric nature was a strength, patient selection and surgical techniques may have varied among the three participating centers, which might influence the results. Considering all participating centers are located in China, the results of our study was only representative of Chinese surgically resectable ICC patients.

In conclusion, the present study demonstrated that both tumor morphology (tumor burden) and tumor-specific biomarker (serum CA19-9) were important when evaluating prognosis of patients with resectable ICC. Serum CA19-9 and TBS showed a synergistic effect on prognostic assessment. The CTC grade, a novel index by combination of TBS and CA19-9, was a promising tool in stratifying ICC patient prognosis following curative resection. Elevated CTC grade was associated with worse prognosis and identified as an independent risk factor for poor OS as well as RFS. Future international multi-institutional studies are needed to validate our results among resectable and unresectable ICC patients.

## Data Availability Statement

The original contributions presented in the study are included in the article/**Supplementary Material**. Further inquiries can be directed to the corresponding authors.

## Ethics Statement

The studies involving human participants were reviewed and approved by the ethics committee of Chongqing University Cancer Hospital and West China Hospital. The patients/participants provided their written informed consent to participate in this study.

## Author Contributions

Conceptualization: HL, DL, GW, and HW. Data curation: HL, RL, HQ, and YH. Formal analysis: HL, WL, and JL. Supervision: DL, GW, and HW. Writing—original draft: HL, RL, HQ, and YH. All authors contributed to the article and approved the submitted version.

## Funding

This work was supported by National Natural Science Foundation of China (81972747, 81672882 and 81870447). We thank Xin Huang from University of Pittsburgh School of Medicine for his support in revising paper.

## Conflict of Interest

The authors declare that the research was conducted in the absence of any commercial or financial relationships that could be construed as a potential conflict of interest.

## Publisher’s Note

All claims expressed in this article are solely those of the authors and do not necessarily represent those of their affiliated organizations, or those of the publisher, the editors and the reviewers. Any product that may be evaluated in this article, or claim that may be made by its manufacturer, is not guaranteed or endorsed by the publisher.

## Supplementary Material

Supplementary Figure 1 Kaplan-Meier curves for 2-year OS **(A)** and early recurrence **(B)** stratified by CTC grade in the derivation cohort. OS, overall survival.

Supplementary Figure 2 Comparison of the predictive value of TBS, CA19-9 and CTC grade in 2-year OS **(A)** and early recurrence **(B)** in the derivation cohort.
