# Supplementary figures and images for: Tumor Burden Score Stratifies Prognosis of Patients With Intrahepatic Cholangiocarcinoma After Hepatic Resection: A Retrospective, Multi-Institutional Study
Source: Front Oncol. 2022 Mar 7;12:829407. doi: 10.3389/fonc.2022.829407 (PMC8940520; doi:10.3389/fonc.2022.829407)

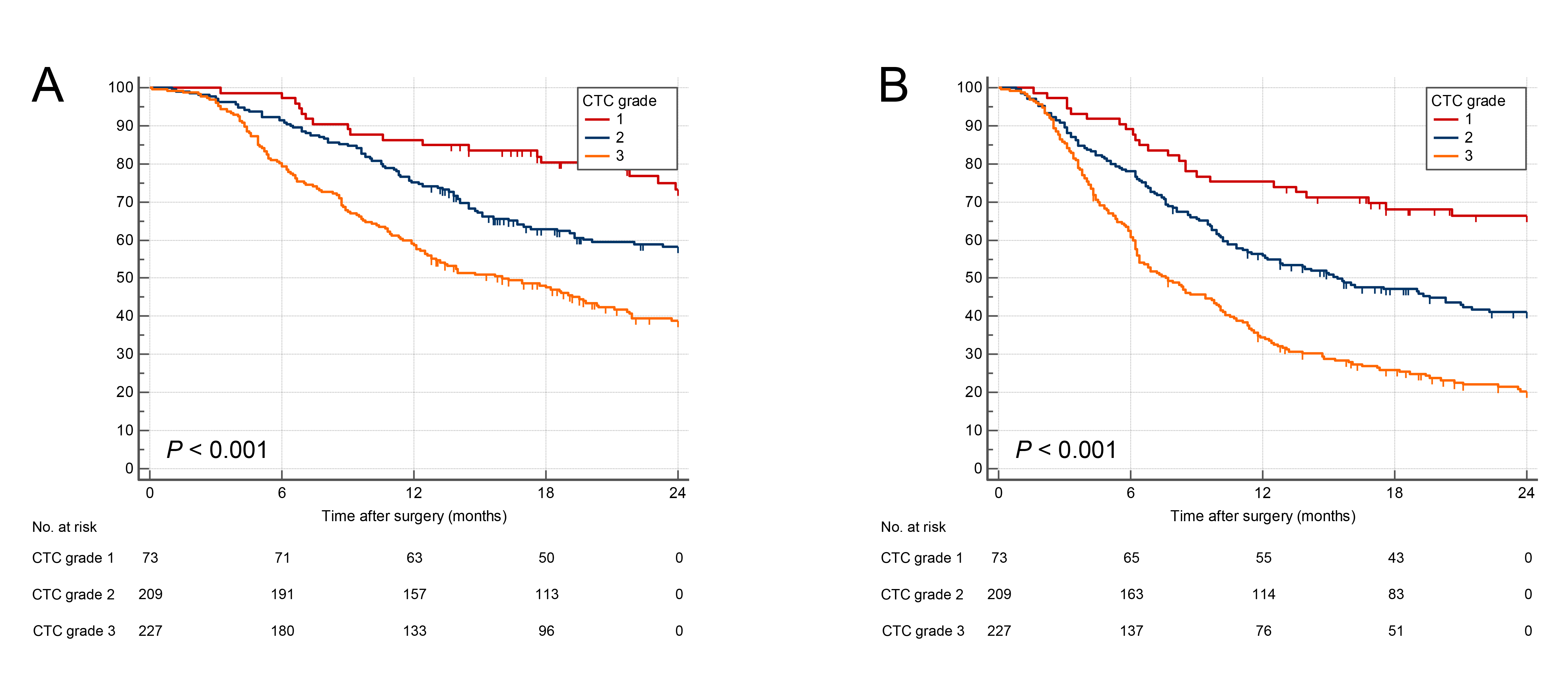

Supplement: Supplementary Figure 1 — Kaplan-Meier curves for 2-year OS (A) and early recurrence (B) stratified by CTC grade in the derivation cohort. OS, overall survival. [file Image_1.jpeg]

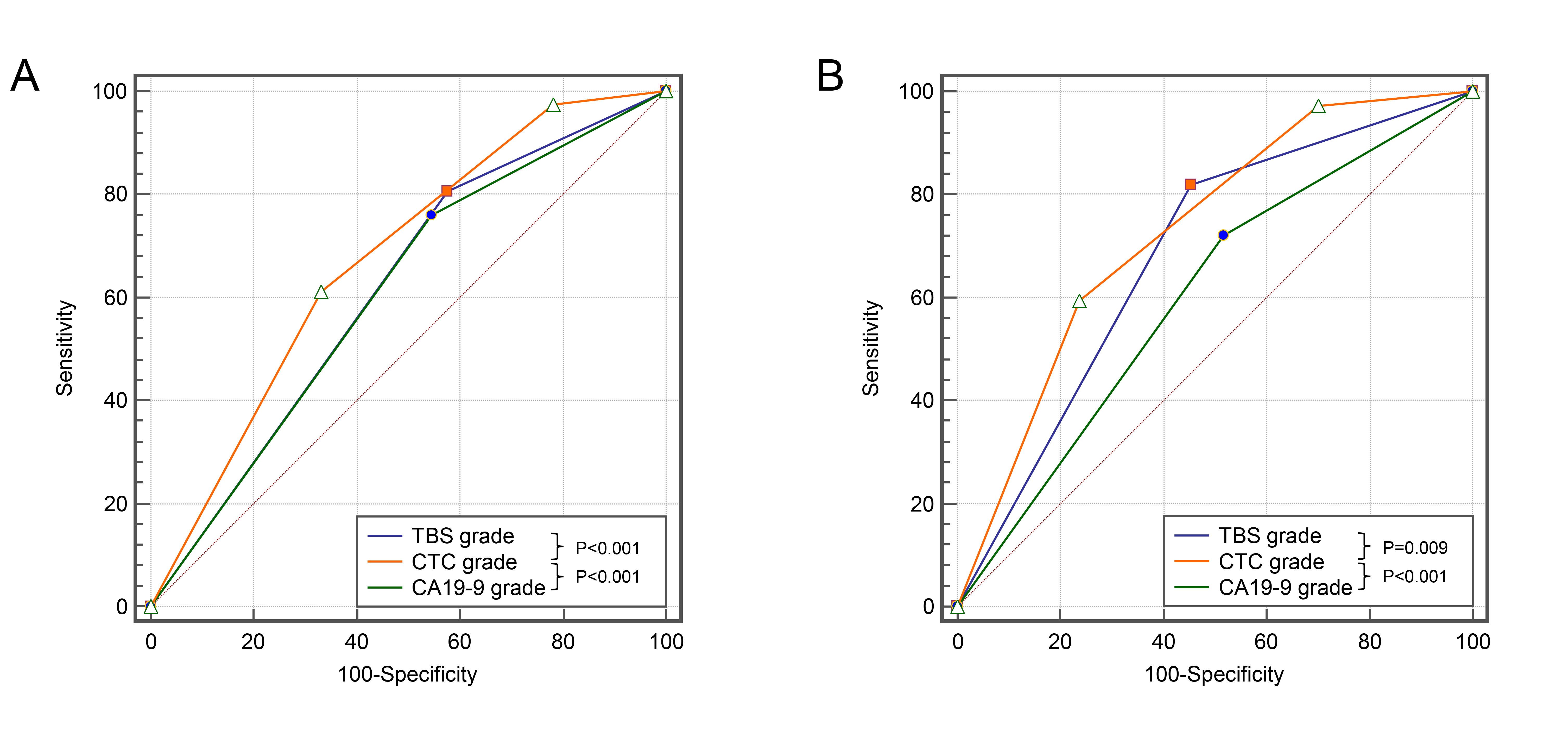

Supplement: Supplementary Figure 2 — Comparison of the predictive value of TBS, CA19-9 and CTC grade in 2-year OS (A) and early recurrence (B) in the derivation cohort. [file Image_2.jpeg]
