# Supplementary material for: Tumor Burden Score Stratifies Prognosis of Patients With Intrahepatic Cholangiocarcinoma After Hepatic Resection: A Retrospective, Multi-Institutional Study
Source: Front Oncol. 2022 Mar 7;12:829407. doi: 10.3389/fonc.2022.829407 (PMC8940520; doi:10.3389/fonc.2022.829407)
Supplement: Supplementary file 3 [file Table_1.docx]

Supplementary table 1. Comparison of predictive value in OS and RFS.

|  | Accuracy | 95% CI | Sensitivity | Specificity |
| --- | --- | --- | --- | --- |
| OS | | | | |
| -CTC grade | 0.688 | 0.646-0.728 | 57.70 | 71.57 |
| -CA19-9 grade | 0.637 | 0.593-0.679 | 75.41 | 51.96 |
| -TBS grade | 0.607 | 0.536-0.649 | 76.72 | 44.61 |
| -Tumor number | 0.581 | 0.537-0.624 | 37.05 | 79.41 |
| -MVI | 0.537 | 0.493-0.581 | 12.79 | 94.61 |
| -Node invasion | 0.606 | 0.562-0.649 | 32.46 | 88.73 |
| -ALRI grade | 0.612 | 0.573-0.652 | 28.31 | 90.59 |
| -CAC grade | 0.675 | 0.634-0.716 | 55.72 | 70.42 |
| RFS | | | | |
| -CTC grade | 0.739 | 0.698-0.776 | 55.98 | 80.14 |
| -CA19-9 grade | 0.637 | 0.593-0.679 | 72.01 | 55.32 |
| -TBS grade | 0.662 | 0.620-0.703 | 77.17 | 55.32 |
| -Tumor number | 0.615 | 0.572-0.658 | 36.68 | 85.82 |
| -MVI | 0.548 | 0.504-0.592 | 12.50 | 97.16 |
| -Node invasion | 0.597 | 0.553-0.640 | 29.35 | 90.07 |
| -ALRI grade | 0.608 | 0.568-0.642 | 31.22 | 83.41 |
| -CAC grade | 0.723 | 0.679-0.752 | 53.31 | 74.45 |

OS, overall survival; RFS, recurrence-free survival; CA19-9, carbohydrate antigen 19-9; TBS, tumor burden score; MVI, microvascular invasion; CTC, combination of TBS and CA19-9 grade; ALRI, aspartate aminotransferase to lymphocyte ratio; CAC, combine ALRI and CA19-9; CI, confidence interval.

Supplementary table 2. Correlation between CTC grade and clinicopathological characteristics in validation cohort.

| Variables | CTC grade | | | P value |
| --- | --- | --- | --- | --- |
|  | 1  (n=25) | 2  (n=50) | 3  (n=66) |  |
| Age, ≤50/>50 | 9/16 | 9/41 | 21/45 | 0.162 |
| Gender, male/female | 15/10 | 22/28 | 35/31 | 0.392 |
| HBsAg, +/- | 5/20 | 17/33 | 20/46 | 0.449 |
| Hepatolithiasis, +/- | 2/23 | 5/45 | 5/61 | 0.922 |
| Tumor size, <5/≥5 | 16/9 | 21/29 | 14/52 | <0.001 |
| Tumor number, 1/2/≥3 | 25/0/0 | 34/8/8 | 41/17/8 | 0.009 |
| Differentiation, well/moderate-poor | 2/23 | 4/46 | 1/65 | 0.242 |
| Capsular invasion, +/- | 14/11 | 29/21 | 48/18 | 0.170 |
| MVI, +/- | 2/23 | 4/46 | 5/61 | 0.996 |
| Node invasion, +/- | 2/23 | 9/41 | 18/48 | 0.024 |
| Perineural invasion, +/- | 0/25 | 6/44 | 9/57 | 0.175 |
| Cirrhosis, +/- | 10/15 | 11/39 | 14/52 | 0.160 |
| TNM stage, I-II/III | 10/15 | 18/32 | 12/54 | 0.038 |
| TBS | 4.07 (1.34) | 6.61 (2.42) | 7.53 (2.15) | <0.001 |

ICC, intrahepatic cholangiocarcinoma; MVI, microvascular invasion; TNM, tumor-node-metastasis; CA19-9, carbohydrate antigen 19-9; TBS, tumor burden score; CTC, combination of TBS and CA19-9 grade.

Supplementary table 3. Cox regression analysis for OS of ICC patients in the validation cohort.

| Variables | Univariate | | | Multivariate | | |
| --- | --- | --- | --- | --- | --- | --- |
|  | HR | 95% CI | *P* | HR | 95% CI | *P* |
| Age, >50/≤50 | 1.246 | 0.777-1.998 | 0.360 |  |  |  |
| Gender, male/female | 0.886 | 0.591-1.330 | 0.560 |  |  |  |
| HBsAg, +/- | 1.326 | 0.862-2.040 | 0.198 | 1.500 | 0.938-2.397 | 0.090 |
| Hepatolithiasis, +/- | 1.150 | 0.578-2.290 | 0.690 |  |  |  |
| Tumor size, ≥5/<5 | 1.593 | 1.022-2.481 | 0.040 |  |  |  |
| Tumor number | 1.448 | 1.127-1.860 | 0.004 |  |  |  |
| Differentiation, moderate-poor/well | 1.546 | 0.748-3.195 | 0.240 |  |  |  |
| Capsular invasion, +/- | 1.400 | 0.906-2.165 | 0.130 | 1.203 | 0.563-2.569 | 0.633 |
| MVI, +/- | 1.737 | 0.898-3.361 | 0.101 | 1.594 | 0.766-3.319 | 0.212 |
| Node invasion, +/- | 2.547 | 1.620-4.003 | <0.001 | 2.212 | 1.231-3.975 | 0.008 |
| Perineural invasion, +/- | 1.764 | 0.979-3.180 | 0.059 | 1.318 | 0.687-2.532 | 0.406 |
| Cirrhosis, +/- | 1.114 | 0.695-1.787 | 0.653 |  |  |  |
| TNM stage, III/I-II | 1.914 | 1.171-3.128 | 0.010 | 1.124 | 0.463-2.731 | 0.796 |
| CA19-9, ≥37/<37 | 1.713 | 1.107-2.651 | 0.016 |  |  |  |
| TBS grade, high/low | 3.116 | 1.867-5.202 | <0.001 |  |  |  |
| CTC grade |  |  |  |  |  |  |
| 1 | Ref. | Ref. | Ref. | Ref. | Ref. | Ref. |
| 2 | 2.016 | 1.201-4.236 | <0.001 | 1.838 | 1.035-3.902 | 0.002 |
| 3 | 4.157 | 2.031-8.511 | <0.001 | 3.413 | 1.623-7.178 | <0.001 |

ICC, intrahepatic cholangiocarcinoma; MVI, microvascular invasion; TNM, tumor-node-metastasis; CA19-9, carbohydrate antigen 19-9; TBS, tumor burden score; CTC, combination of TBS and CA19-9 grade; OS, overall survival.

Supplementary table 4. Cox regression analysis for RFS of ICC patients in the validation cohort.

| Variables | Univariate | | | Multivariate | | |
| --- | --- | --- | --- | --- | --- | --- |
|  | HR | 95% CI | *P* | HR | 95% CI | *P* |
| Age, >50/≤50 | 0.988 | 0.640-1.527 | 0.958 |  |  |  |
| Gender, male/female | 0.935 | 0.635-1.376 | 0.732 |  |  |  |
| HBsAg, +/- | 1.395 | 0.994-2.250 | 0.054 | 1.460 | 0.902-2.364 | 0.123 |
| Hepatolithiasis, +/- | 0.999 | 0.504-1.981 | 0.997 |  |  |  |
| Tumor size, ≥5/<5 | 2.144 | 1.379-3.335 | 0.001 |  |  |  |
| Tumor number | 1.586 | 1.243-2.023 | <0.001 |  |  |  |
| Differentiation, moderate-poor/well | 2.139 | 1.072-4.268 | 0.031 | 1.499 | 1.085-4.800 | 0.278 |
| Capsular invasion, +/- | 1.486 | 0.976-2.264 | 0.065 | 1.126 | 0.526-2.410 | 0.761 |
| MVI, +/- | 2.054 | 1.067-3.955 | 0.031 | 2.226 | 1.063-4.660 | 0.034 |
| Node invasion, +/- | 1.927 | 1.225-3.034 | 0.005 | 1.509 | 1.095-2.081 | 0.012 |
| Perineural invasion, +/- | 1.486 | 0.829-2.664 | 0.183 | 1.130 | 0.575-2.218 | 0.723 |
| Cirrhosis, +/- | 0.926 | 0.582-1.474 | 0.747 |  |  |  |
| TNM stage, III/I-II | 1.889 | 1.184-3.014 | 0.008 | 1.387 | 0.580-3.317 | 0.462 |
| CA19-9, ≥37/<37 | 1.530 | 1.016-2.305 | 0.042 |  |  |  |
| TBS grade, high/low | 3.133 | 1.931-5.082 | <0.001 |  |  |  |
| CTC grade |  |  |  |  |  |  |
| 1 | Ref. | Ref. | Ref. | Ref. | Ref. | Ref. |
| 2 | 1.971 | 1.008-3.513 | 0.021 | 1.748 | 1.115-2.741 | 0.015 |
| 3 | 3.742 | 1.939-7.221 | <0.001 | 3.175 | 1.600-6.299 | 0.001 |

ICC, intrahepatic cholangiocarcinoma; MVI, microvascular invasion; TNM, tumor-node-metastasis; CA19-9, carbohydrate antigen 19-9; TBS, tumor burden score; CTC, combination of TBS and CA19-9 grade; RFS, recurrence-free survival.

**Supplementary figure legends**

**Supplementary figure 1.** Kaplan-Meier curves for 2-year OS (A) and early recurrence (B) stratified by CTC grade in the derivation cohort. OS, overall survival.

**Supplementary figure 2.** Comparison of the predictive value of TBS, CA19-9 and CTC grade in 2-year OS (A) and early recurrence (B) in the derivation cohort.
